# A Case of Suicidal Perforation of the Stomach by a Red-Hot Iron, with Remarks

**Published:** 1888-06

**Authors:** Lockhart Stephens

**Affiliations:** Emsworth (late House Surgeon to the Bristol General Hospital)


### Clinical Records.

---

A CASE OF SUICIDAL PERFORATION OF THE STOMACH BY A RED-HOT IRON, with Remarks. By LOCKHART STEPHENS, M.R.C.S., Emsworth (late House Surgeon to the Bristol General Hospital).

S. W., æt. 35, by trade a journeyman nailer. Admitted to the Bristol General Hospital on June 9th, 1885, at 6 p.m., under the care of Mr. Dobson, to whose kindness I am indebted for permission to publish the case.

The following history was obtained:—About two months before Christmas patient went to an Asylum at Reading, and was discharged, cured, at Christmas. He suffered from melancholia, but had never before attempted suicide. On June 9th, whilst at work forging nails, he was seen with a red-hot iron about two feet in length, the cool end of which he had against the wall, and the heated end against his belly. The iron was so hot that it ignited his apron. One of his mates ran up, gave him a push, and made him drop the iron. He said that if he was allowed to go on with his work he should be all right, and he was allowed to resume work. The iron rod was a thin one, used in making horse nails. Not long after this first attempt, he made the iron white-hot, rushed by the man who had previously stopped him in the former attempt, and again thrust the iron against his belly. The iron entered about four or five inches, according to the man who pulled it out. He was then placed on a stretcher and brought to the Hospital.

On admission, June 9th, 6 p.m., patient was suffering from severe shock; face very pale; forehead covered with large beads of perspiration; respirations very feeble, mainly thoracic in character; could answer questions; said he was not in pain. His clothes were found to be burned through, in a hole about level of umbilicus; and when removed, his shirt and undervest were stained with blood, roughly estimated at ʒij. or ʒiij. Pulse scarcely felt; extremities cold.

Immediately to right side of umbilicus, and almost entering upon right margin of that structure, was a wound, edges scorched and jagged, having the peculiar odour of burnt flesh; it measured about $\frac{1}{3}$ inch in every diameter: thin bloody serum oozed slightly from the wound, but soon stopped.

6.15 p.m. Patient was at once placed in bed, lying flat, head low. As the pulse could scarcely be felt, 20 minims of brandy were given subcutaneously. Pulse quickened from 60 to 80 per minute. He then belched up some wind, and shortly vomited. Still very pale and sweating; skin cold and clammy. On passing hand gently over epigastrium, he experienced great pain. P. 72, very feeble. R. 9, shallow and feeble. Eyelids half closed, pupils widely dilated; roused up when spoken to. T. 98.2°. The symptoms gave no clue to the viscus injured, and as the man was much too feeble to admit of any operative interference, the wound was dressed with boracic lint soaked in carbolic lotion (1 in 20).

6.50 p.m. P. 64, very feeble again. R. 11. Ordered 15 minims of tinct. opii in brandy, $\frac{1}{2}$ oz. every four hours.

8.0 p.m. Seems weaker, as if dying. Is sensible; said he would like to see his wife. Seen by the clergyman of his parish, to whom he repeated a statement which he had made some weeks previously, to the effect that he was a scoundrel, and had been unfaithful to his wife; whereas he was a good husband, and had a small sum of money in the savings' bank. Says he is in less pain. Restless, and rolling from side to side if not prevented by nurse. Has not vomited for 15 or 20 minutes.

10.0 p.m. Constant eructation since 8.0 p.m. Vomited some brownish watery mucus about 9.30. Still very pale and sweating. P. 70, but very feeble. Respiration jerky. Gas heard coming through wound, showing perforation of bowel. He looks as if he might die at any moment.

June 10th. During the night he has been quieter; just alive and no more. Pulse can now scarcely be felt; is much weaker. Respirations very feeble indeed. Wound dressed. Gas again heard coming through wound, during dressing: odour not feculent.

1.20 p.m. Sank gradually, and died.

*Post-mortem Examination.*—June 11th, 1885. 22½ hours after death. Weather warm and dry. No signs of decomposition.

Rigor mortis marked in all joints. Hypostasis in back, buttocks, shoulders, and arms down to elbows.

Body that of a well-nourished and well-developed man, about 5 feet 9 inches in height. No abdominal distension.

In median line of anterior surface of body are two eschars: the *upper* one situated over lower part of sternum, beginning at ¼ inch from tip of ensiform cartilage, and being there ⅛ inch in width; extending upwards in median line for 2 inches, where it was ¾ inch in width; then tapering off to a scratch towards left side for another inch. The eschar involved the *skin*, but not the subcutaneous connective tissue, and was the result of the first attempt at suicide.

The *lower* one was the aperture of entrance of the hot iron. It was situated immediately to right side of umbilicus; the edges were jagged and scorched; measuring $\frac{1}{4}$ inch in right oblique and $\frac{1}{2}$ inch in left oblique diameters respectively from above downwards. It was *partially* surrounded by an eschar, which extended in median line 1 inch above and 2 inches below it, $1\frac{1}{2}$ inches in left oblique diameter, and not at all to the right side.

The abdomen was then opened by incisions from tip of ensiform cartilage to pubes, so as to leave the wound and umbilicus *in situ*. At the lower part of the abdomen coils of small intestine were found, acutely inflamed to the extent that their natural lustre was dull; but lymph had not formed, nor were they adherent to one another. They were bathed on their deep aspect in dark blood, which occupied all the dependent parts of the abdominal cavity, and of which 42 ozs. were removed and measured.

The great omentum lay over the viscera as far down as the umbilicus.

On separating the parts, which were covered here with black grumous material and dark semi-clotted blood, there were seen to be two clean-cut holes in the stomach, from which the blackish material, partly stomach-contents, and blood had come, the latter being here in larger quantity than elsewhere.

From the large quantity of blood found in the peritoneal cavity, it appeared probable that some large vessel had been wounded: with great care, therefore, the diaphragm, liver, and abdominal viscera were removed *en masse* and examined. The aorta and vena cava inferior were very carefully dissected, and were all found to be intact. The bleeding, therefore, must have come from the wounds in the stomach, which on further examination proved to be correct.

*The stomach* showed two wounds. (*a*) Situated exactly on the inferior border, 2½ inches from the pyloric orifice, square in shape, measuring ½ inch in all diameters, and corresponding to the shape of the piece of iron used. The vessels on this border of the stomach were involved in the wound. This wound was the point of entrance of the iron.

(*β*) Situated on the anterior surface, immediately below the lesser curvature, 2 inches from the pyloric orifice, oblong in shape, ¾ inch by ½ inch. The mucous membrane was projecting through from within out. On dissecting out the vessels, two large branches of the *artery*—the right gastro-epiploic and pyloric branches of the hepatic artery—with its accompanying veins, ran into the upper margin of this wound.

The other organs throughout were healthy, and beyond noting their respective weights, left nothing to be recorded.

Liver, 44 oz.

Heart, 9½ oz. Quite empty.

Lungs—Right, 19 oz. } a little congested at bases.

Left, 14 oz. }

Kidneys, each 5 oz.

Spleen, 3 oz.

Brain, 52 oz. { To naked eye and on section it appeared quite normal.

*Remarks.*—This case is worthy of record both from a surgical and medico-legal point of view: the former, bearing on the nature of the injury and line of treatment to be adopted in such cases; the latter, from the rarity of such a means of self-destruction, and the difficulties which might have arisen had the body been found under circumstances which did not clearly point to suicide.

In the outset I must say definitely that at no period of the man's life did his condition, in my judgment, admit of operative interference; and in this I am supported by my friend, Mr. W. J. Penny, who saw the case on admission.

When admitted, the patient was suffering from severe *shock*, apparently the result partly of the injury inflicted, and partly of the condition of mind which led to the act. This shock, at first due to the above-named causes, was later on increased by the hæmorrhage found after death, but which I failed to appreciate during life.

Mr. Dobson, under whose care the case was admitted, but who did not see the case during life, was of opinion that abdominal section might have been done, being an advocate of the practice of early abdominal section in cases of injury of abdominal viscera and in intestinal obstruction. I may here allude to a communication by Mr. Dobson to the *Bristol Medico-Chirurgical Journal*, vol. i, 188, in which he suggests the advisability of operative interference in gastric ulcer with severe hæmatemesis. In such cases, however, it should be borne in mind, the course open to the surgeon is to a certain extent clear; for he knows the organ with which he has to deal, and within certain limits also the nature of the disease which he has to treat. Cases such as that which I have reported are, I think, not entirely analogous; for the surgeon does not know the extent or nature of the injury, or hardly the organ involved: so that to perform abdominal section for gastric ulcer would seem to be a comparatively straightforward matter, when placed beside a case of indefinite though very severe injury to some viscus not definitely known.

After a somewhat careful search through text-books and journals, I can find no case precisely similar as regards the means whereby the injuries were inflicted. Stabs and bullet-wounds cut and tear the abdominal wall and viscera, but do not *destroy* in their course as a red-hot iron would do; and although at first sight one would have thought, as both Mr. Penny and myself remarked at the time, we ought to have no fear from hæmorrhage, because of the cauterizing effect of the hot iron; yet the *post mortem* examination showed that had any operative treatment been attempted, the source of hæmorrhage would have been one of the chief difficulties to have been overcome.

Coming now to the information to be gained from the *post mortem* examination: What light does this case throw on the great question of operation in abdominal injuries? To have opened this man's abdomen, using the aperture of entrance of the iron as the centre of an incision three or four inches long, the first thing reached would have been the great omentum, covering up the structures beneath: the transverse colon lay next to hand; and above it, somewhat out of reach of the incision, certainly above its upper extremity, was the stomach.

Supposing the stomach had been seized, and two holes, not mere cuts but holes, seen—what should have been done? I take it, the upper one should have been closed by continuous suture, and the lower one brought to the abdominal wall as in gastrostomy.

The vessels of the stomach being involved in both wounds, introduced another difficulty. From the amount of blood found in the peritoneum, I expected to find some other larger vessel wounded. The presence of the two wounds in the organ may be explained by the organ being partly distended at the time, and the probability that the hot iron bar was thrust in obliquely, and not vertically, to the abdominal wall.

The report of this case will form one link—perhaps a weak one—in the very long chain of information which we want before rules can be laid down as to the line of treatment to be adopted in such cases. For the present I apprehend that each case has to be judged entirely on its own merits, the main points to be attended to being: the means whereby the injury was inflicted, the nature of the injuries as nearly as we can judge, the condition in which we find the patient, and the probability of success if we operate; this last depending mainly on the other three.

[N.B.—The writer's remarks on this case were written in 1885, and he has no hesitation in saying that bearing in mind recent experience in such cases, he would at once perform abdominal section.—L. S.]

---

